# Visibility of medical informatics regarding bibliometric indices and databases

**DOI:** 10.1186/1472-6947-11-24

**Published:** 2011-04-15

**Authors:** Cord Spreckelsen, Thomas M Deserno, Klaus Spitzer

**Affiliations:** 1 Institute for Medical Informatics, RWTH Aachen University, Aachen, Germany

## Abstract

**Background:**

The quantitative study of the publication output (bibliometrics) deeply influences how scientific work is perceived (bibliometric visibility). Recently, new bibliometric indices and databases have been established, which may change the visibility of disciplines, institutions and individuals. This study examines the effects of the new indices on the visibility of Medical Informatics.

**Methods:**

By objective criteria, three sets of journals are chosen, two representing Medical Informatics and a third addressing Internal Medicine as a benchmark. The availability of index data (index coverage) and the aggregate scores of these corpora are compared for journal-related (Journal impact factor, Eigenfactor metrics, SCImago journal rank) and author-related indices (Hirsch-index, Egghes G-index). Correlation analysis compares the dependence of author-related indices.

**Results:**

The bibliometric visibility depended on the research focus and the citation database: Scopus covers more journals relevant for Medical Informatics than ISI/Thomson Reuters. Journals focused on Medical Informatics' methodology were negatively affected by the Eigenfactor metrics, while the visibility profited from an interdisciplinary research focus. The correlation between Hirsch-indices computed on citation databases and the Internet was strong.

**Conclusions:**

The visibility of smaller technology-oriented disciplines like Medical Informatics is changed by the new bibliometric indices and databases possibly leading to suitably changed publication strategies. Freely accessible author-related indices enable an easy and adequate individual assessment.

## Background

### Introduction

*Bibliometrics *is defined as "the scientific and quantitative study of publications" [1]. *Bibliometric indices *quantify the scientific impact of journals, research institutions, or scientists by a statistical analysis of the publication effort - mainly by analysing citations [2,3]. *Bibliometric visibility *of scientific research measures how scientific work in the respective field is perceived and valued.

In the last years, online-tools like ISI Web of Knowledge, the SCImago Journal & Country Rank Portal, Google Scholar, and GoPubmed have dramatically improved the availability of bibliometric information [4,5]. At the same time, the misuse of bibliometrics - which triggered criticism for a long time - has become a major concern [6-8]. In this situation, new bibliometric scores have been developed, are now going to be established, and may in near future rapidly affect the relative bibliometric visibility of research fields.

Bibliometrics can be applied to explore research trends and the conceptual structure of research fields. This has been done for Medical Informatics (MI) with some remarkable results: A pioneering intercitation analysis yielded that MI has a special core literature structured by major focus areas [9]. Bansard et al. investigated the relation between MI and Bioinformatics showing that "these domains are still relatively separated" [10]. DeShazo et al. characterized the increasing output of MI using the frequencies of corresponding Medical Subject Headings (MeSH) terms [1]. Finally, a cluster analysis of titles and abstracts of MI literature showed that MI research can be mapped to three different subdomains [11]. A recent study shows that new distinct subfields have emerged in the last years representing the growing influence of the Internet and of organisational and user driven perspectives [12].

The most influential use of bibliometric measures is the assessment of the scientific impact of journals, institutions, or individual researchers. Funding, staffing, and individual careers are influenced by bibliometric indices, although, for instance, the application of the ISI Journal impact factor for assessment of individuals or institutions is widely regarded as inadequate [7]. But, given that "the scientific community will be doomed to live by the numerically driven motto, 'survival by your impact factors'" [7], it is important to investigate how the choice of different bibliometric indices affects the bibliometric visibility.

### Literature Review

Bibliometric measures can be subsumed to three major categories: indices 1) rating journals 2) rating authors and 3) rating individual publications. In the following, we focus on indices that are already available.

#### Indices rating journals

The *Journal Impact Factor *(JIF) was established in the 1960s and has been the most influential measure over the last decades [2,13]. The JIF is an unbounded, positive rational number calculated by taking the number of citations to articles published by a specific journal in the previous two years and dividing this by the number of articles published by the journal during the same interval.

The more recently introduced *Eigenfactor metrics *achieved an importance-based weighting of citations by an approach similar to the PageRank Algorithm of Google [8,14]. In principle, the metrics estimates the fraction of time a reader would spend on average at a specific journal when taking an infinite random walk through the literature following the chain of citations. The metrics is based on the calculation of the leading eigenvector of a stochastic matrix derived from the cross-citation matrix of the journals considered. The approach specifies two indices:

1. The *Eigenfactor Score *(ES) is a "measure of the journal's total importance to the scientific community" [8]. It aggregates the contributions of all articles contained in the journal to the random walk described above and thus scales with the size of the journal.

2. The *Article Influence Score *(AIS) characterizes the journal's impact by measuring the mean influence of an average article. An AIS above or below 1.0 indicates that papers published in the respective journal have an influence above or below average, respectively.

The *SCImago Journal Rank *(SJR) also adopts the PageRank approach. SJR is calculated by iteratively accounting for the "prestige of a journal" [15], which is constituted by the journal's papers being highly cited in other prestigious journals.

The *Hirsch-index *(HI) originally introduced in 2005 as a means to rate scientific authors is therefore explained in detail in the next section [16]. However, the approach is applicable to journals as well. This is done, for instance, by the SCImago Journal & Country Rank Portal (see Table 1 below).

**Table 1:** Index data available from citation databases

Index	ISI/Thompson Reuters	Scopus	Google Scholar
Journal Impact Factor (JIF)	X		
Eigenfactor Score (ES)	X		
Article Influence Score (AIS)	X		
SCImago Journal Rank (SRJ)		X	
Journal Hirsch Index (Journal HI)		X	
Author Hirsch Index (Author HI)	X	X	X
g-Index (GI)	S	S	X
Google Page Rank (GPR)			X

(X: directly provided by the corresponding web portal, S: accessible by sorting of results)

#### Indices rating authors

A scientist achieves a Hirsch index (HI) of h, "if *h *of his or her *N*_*p *_[published] papers have at least *h *citations each and the other (*N*_*p *_- *h*) papers have <= *h *citations each" [16]. The HI avoids focusing on productivity only, it does not rely on arbitrary limits, and it is neither inflated by a few highly cited review articles nor by a huge amount of co-authored papers. HI reflects seniority, i.e. it would grow for a while after a researcher has delivered considerable work to the scientific community, even if the researcher did not provide further scientific contribution.

In 2006, L. Egghe smoothed this criterion by defining the *g-index *(GI): "Given a set of articles ranked in decreasing order of the number of citations that they received, the g-index is the (unique) largest number such that the top *g *articles received on average at least *g *citations" [17]. In comparison, GI needs more calculation steps than HI and decreases the influence of highly-cited articles.

#### Indices rating papers

There are only very few approaches to rank individual publications. A basic measure is the total number of citations received by a paper. It is implicitly applied when rewarding highly cited papers. The total number of citation is time-dependent (on average an older paper is cited more often than a recent one) and the effect of citations by low-interest or even low-quality papers is likely to be greater than in the case of journals, where cross-citations average over individual papers. Except for indicating top ranking, highly cited papers the total number of citations is not reported to be regularly applied.

The literature reports approaches, which rely on *Google's Page Rank *(GPR) *Index *and which are applied to website publishing [18,19].

### Objectives

The purpose of this study was to investigate whether the new bibliometric approaches may change the relative bibliometric visibility of MI research compared to a high-impact benchmark, and (eventually) determine the strength of this effect. MI is a technology-oriented multidisciplinary domain. Compared to classical medical disciplines MI is a relatively small field attracting less attention. Nonetheless, while often being affiliated with medical departments or medical schools, MI has to compete with these disciplines for attention and funding. Therefore, MI is an exemplary field where changes of the bibliometric visibility are of vital interest.

The study addresses the following questions:

1. Which sets of journals should be chosen to represent the research activity of MI and to form a benchmark representing a high-impact field?

2. Are there differences in the visibility of MI as measured by bibliometrics scores caused by the transition to the new bibliometric indices?

## Methods

### Terminology

The total *publication output *of a research field is practically impossible to determine. Therefore, it has to be estimated from a representative subset of publications referred to as *corpus*.

*Bibliometric indices *(see above) are calculated using a *citation database*, defined as a database retrieving publications by which a given publication is cited. If the database included only a small fraction of publication output of a given field, the respective index would not adequately measure the relevant research activity in that domain. In order to quantify this aspect, *index coverage *is defined here as the fraction of a given corpus for which index data is available. An *index score *is a (suitable) aggregate measure of the publication output based on the index values and the coverage.

Thus, *bibliometric visibility *is indicated by the index score based on the index coverage. Index score and coverage depend on a) the index, b) the database used for index calculation, and c) the corpus representing the publication output.

Table 1 shows an overview of the indices provided by the respective citation databases (i.e. the global availability of indices).

### Computing Tools

All statistical analyses were performed using SPSS, version 16.0 (SPSS Inc., Chicago, IL, USA). Table 2 shows the bibliometric tools and resources used for this study. With the exception of the *ISI Web of Science *all of them are publicly available. The *ISI Web of Science *is nonetheless included because of its widespread use by academic institutions.

**Table 2:** Bibliometric tools and sources

Tool			Database		Task
Name	URL	Type	Name	Type	
GoPubmed	http://www.gopubmed.com/	search interface	Pubmed/Medline	bibliographic	- rank journal corpus (based on MeSH terms)- rank author corpus (based on number of publications)

ISI Web of Science	http://www.isiknowledge.com/	web portal	ISI/Thompson Reuters	citation	- retrieve JIF, ES, AIS for journals- determine HI(ISI) for authors
QuadSearch	http://quadsearch.csd.auth.gr/	metasearch engine	Google Scholar	citation	- retrieve HI(Scholar) and GI(Scholar) for authors (based on Google Scholar citation data)
SCImago Journal & Country Rank Portal	http://www.SCImagojr.com/	web portal	Scopus	citation	- retrieve SRJ and HI(SCI) of journals

NCBI Journals database	http://www.ncbi.nlm.nih.gov/sites/entrez?db=journals	search interface	NCBI	table of names	- standardize journals names

### Journal corpora and high-impact authors

As a prerequisite of this study journals and authors representing the realm of MI research need to be chosen. Four criteria guided the selection of a set of journals (journal corpus) meant to characterize MI:

1) The corpus only includes peer-reviewed journals.

2) These journals publish latest MI research, which is indicated if at least one original paper attributed to MI was published in the most recent issue.

3) Objective and reproducible selection criteria are used for defining the corpus, which preferably are based on bibliometric measures.

4) The corpus or at least the selection criteria should have been previously proposed by other researchers and approved by peer-reviewing, which requires a related article being published in a peer-reviewed journal. Therefore, this study should compare corpora previously acknowledged to represent Medical Informatics.

Bibliometric studies *starting from *a given set of core journals of a field rather than defining such a journal selection violate criterion three. Also, the corpora previously defined by Morris & McCain and Bansard et al. did not fully meet the criterion of objectivity and were therefore excluded [9,10].

#### Schuemie corpus

In contrast, Schuemie et al. started with the journals assigned to the ISI category "Medical Informatics" [11]. Based on a statistical similarity measure they retrieved additional journals similar to this seed set, and iteratively formed a set of 16 MI journals with a high respective similarity. Appendix 2 gives a more detailed description of the procedure. In the following, we will refer to this collection as Schuemie corpus (see Appendix 3, Table 3).

**Table 3:** Overview of the journal corpora

**No**.	Journal name according to NCBI	Schuemie	MeSH-MI	MeSH-Med
1	Acad Med			x
2	Am J Gastroenterol			x
3	Am J Med			x
4	Ann Intern Med			x
5	Artif Intell Med	x		
6	Bioinformatics		x	
7	BMC Med Inform Decis Mak	x		
8	Br Med J		x	
9	Can J Cardiol			x
10	Circulation			x
11	Clin Cardiol			x
12	Comput Biol Med	x		
13	Comput Inform Nurs	x		
14	Comput Methods Programs Biomed	x		
15	Gastroenterology			x
16	Health Data Manag		x	
17	Health Manag Technol		x	
18	Healthc Inform		x	
19	IEEE Trans Inf Technol Biomed	x		
**20**	**Int J Med Inform**	**x**	**x**	
**21**	**Int J Radiat Oncol Biol Phys**		**x**	**x**
22	J AHIMA		x	
23	J Am Coll Cardiol			x
**24**	**J Am Med Inform Assoc**	**x**	**x**	
25	J Biomed Inform	x		
26	J Clin Oncol			x
27	J Gen Intern Med			x
28	J Med Internet Res	x		
29	J Med Syst	x		
30	J Rheumatol			x
31	Med Inform Internet Med	x		
32	Med Phys		x	
33	Medinfo	x		
**34**	**Methods Inf Med**	**x**	**x**	
35	MIE Procs*	x		
36	Mod Healthc		x	
37	Nephrol News Issues			x
38	Nucleic Acids Res		x	
39	Phys Med Biol		x	
40	Proc AMIA Symp	x		
41	Proteins		x	
42	Radiother Oncol		x	
43	Rheumatology			x
44	Stat Med		x	
45	Stud Health Technol Inform		x	

	(* *not included in NCBI Journals)*	n = 16	n = 18	n = 15

Journals belonging to more than one corpus are highlighted (bold face font).

#### MeSH-MI corpus

DeShazo et al. defined a body of MI literature selecting papers assigned to the MeSH term "Medical Informatics" or its subcategories (MeSH-MI papers) [1]. They further investigated journals that at least have published 20 MeSH-MI papers. DeShazo et al. do not investigate whether a journal continuously publishes a considerable number of MeSH-MI papers over the years. Hence, some modifications are made: Based on the criterion of DeShazo we retrieved the (top-50) journals publishing the most MeSH-MI papers annually since 1987 and selected only those journals, which have sustained a top-50 rank for at least 8 of the last 10 years [20]. This yielded a set of 18 MI journals referred to as MeSH-MI corpus in the following (see Appendix 3, Table 3).

#### MeSH-Med corpus

For comparison, a third corpus is defined. While frequently being part of medical institutions MI directly competes against medical specialities. Therefore, we identified medical fields known to achieve high bibliometric scores by

1) selecting the 45 (out of 172) ISI categories referring to medical specialities,

2) retrieving the ISI category summary list, and

3) ranking the list by the product of the median and the aggregate impact.

Since the MeSH term "Internal Medicine" directly subsumes both high-scoring fields within the MeSH classification hierarchy ("Medical Oncology" and "Hematology"), we selected Internal Medicine as field of research (52,434 papers). Using the same approach as in the case of the MeSH-MI a corpus of 15 medical journals was defined (see Appendix 3, Table 3), which is referred to as MeSH-Med corpus.

#### High-impact authors

In order to calculate author-related indices, the authors of the 10,000 papers published most recently in a specific corpus were selected and ordered by the number of authored papers using GoPubmed. The author-related bibliometric indices were further investigated for the top-25 authors. The HI was obtained from the ISI Web of Science by running a search for an author and opening the respective citation report.

### Similarity measures and distance metrics

#### Cosine similarity

The similarity of corpora is based on *MeSH profiles*: The list of top-50 MeSH terms most frequently used for indexing papers of the corpus was selected using GoPubmed and ordered by the frequency of each term (considering the 100,000 papers published most recently). The resulting vectors of the frequency rates were used to calculate the cosine similarity (see Appendix 4). Additionally, the MeSH profiles were inspected qualitatively by reading the terms and identifying concepts, which describe the aspects, these terms have in common (e.g. "DNA", "Genes", "Sequence alignment" could be summarized by the concept "Molecular Biology").

#### Bibliographic visibility

Based on the ratings of 2007, the JIF, ES, SJR, and HI were retrieved for all journals contained in the three corpora. The *overall bibliographic visibility *of a research field (e.g. MI) is defined here by summing up the scores of the corresponding corpus on a per-paper-basis. In other words, a corpus is considered as the scientific output of the field of research and treated exactly as if the research field was an individual researcher.

#### Aggregate score

The aggregate score is meant to measure the scientific impact on a per-paper-basis. Let denote the total number of papers in the journal *j *of corpus *C *(i.e. Schuemie, MeSH-MI, or MeSH-Med), which additionally fulfill a given selection criterion (*crit*), and *b*_*j *_denote the bibliometric index of the journal *j*. Then, the following sum yields the total aggregate score(1)

The average score *a *is defined as(2)

## Results

### Characteristics of the journal sets (journal corpora)

**Figure 1:**
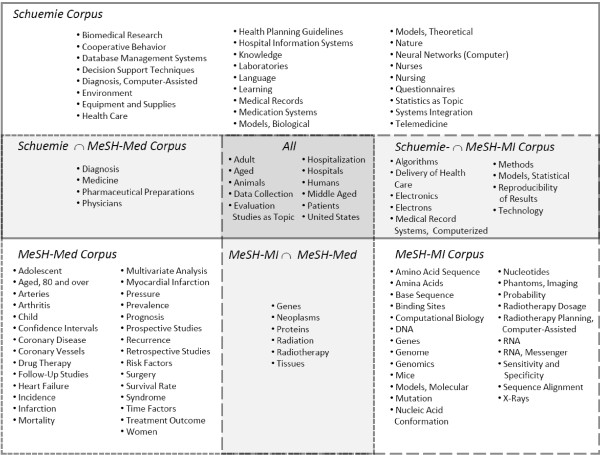
**Overview of the MeSH-profiles of the three sets of journals**. The white, light gray, and dark gray areas contain the MeSH terms used exclusively for one corpus, found in the corresponding intersection of two profiles, and assigned to all corpora, respectively.

The Schuemie corpus consists of 16 journals; the MeSH-MI corpus contains 18, the MeSH-Med corpus 15 journals (Appendix 3, see Table 3). Three journals belong to both MI-related corpora (*Int J Med Inform, J Am Med Inform Assoc, Methods Inf Med*), one journal belongs to the MeSH-MI and the MeSH-Med Corpus (*Int J Radiat Oncol Biol Phys*). There are no other intersections. The comparison of the MeSH-profiles yielded (i) index terms specific for each corpus (Schuemie: 26 terms, MeSH-MI: 22 terms, MeSH-Med: 29 terms), (ii) pairwise intersections, and (iii) a core set of 11 terms found in all three profiles (Figure 1).

The cosine similarities of the corpora are shown in Table 4.

**Table 4:** Cosine similarity of the journal corpora based on the MeSH profiles.

	MeSH-MI	Schuemie	MeSH-Med
MeSH-MI	1.0	.17	.18
Schuemie		1.0	.20
MeSH-Med			1.0

Range: 0 - "least similar", 1 - "most similar"

Only three authors were contained in more than one top-25 list: "Bakken S", "Bates D", "Haux R" (all in MeSH-MI and Schuemie).

### Coverage of the bibliometric databases

**Figure 2:**
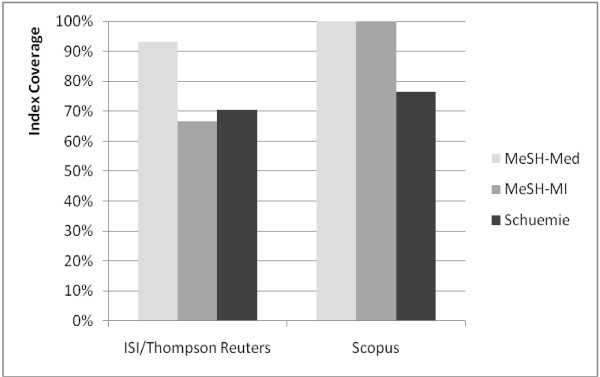
**Index coverage of the bibliometric databases**. The diagram shows the percentage of journals included in the ISI Journal Citations Report vs. the SCImago Portal.

The rate of journals covered by the two most relevant bibliometric databases (ISI/Thompson Reuters vs. Scopus citation data) differed manifestly when comparing different corpora and different bibliometric databases (Figure 2). Google Scholar was not included, because it covers web publications instead of journals.

The MeSH-MI and the MeSH-Med corpus profit most from the improved coverage of Scopus: 100% coverage, compared to 76.5% (Schuemie corpus), whereas the maximum coverage found in the ISI database was 93.3% (MeSH-Med), followed by 70.6% (Schuemie), and 66.7% (MeSH-MI).

### Effects of the journal-related bibliometric indices

**Figure 3:**
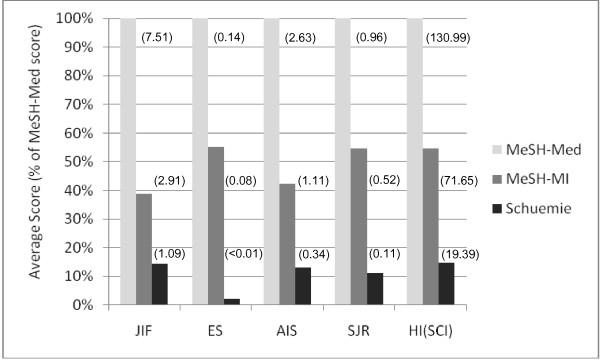
**Percentage of the average scores *a *of the MI-related corpora relative to the MeSH-MI score calculated for different bibliometric indices**. The absolute value of *a *is given in brackets.

The average score *a *as defined in (3) was calculated for JIF, ES, AIS, HI(SCI), and SJR. The comparison of the different indices was enabled by expressing the scores reached by MeSH-MI and Schuemie corpus as a percentage of the MeSH-Med-based benchmark (Figure 3). Irrespective of the bibliometric indices, all MI-related corpora scored below 56% of the medical corpus (MeSH-Med).

For JIF, AIS, SJR, and HI(SCI) the MeSH-MI scores were 2.7, 3.3, 3.7 and 4.9 times greater than the Schuemie score, respectively. In case of ES, the Schuemie corpus was completely marginalized. Compared to the JIF, the MeSH-MI corpus gained about 40% of relative scoring when using the ES, HI(SCI), or SJR. The proportion remained more stable for AIS (relative gain: 9%).

In contrast, the Schuemie corpus lost about 85%, 11%, and 22% of relative scoring using ES, AIS, and SJR, respectively. Only in the case of HI(SCI) the proportion remained almost unchanged. Thus, as a key result of this study, the two MI specific corpora performed differently when the new bibliometric indices were applied.

### Effects of the author-related bibliometric indices

**Figure 4:**
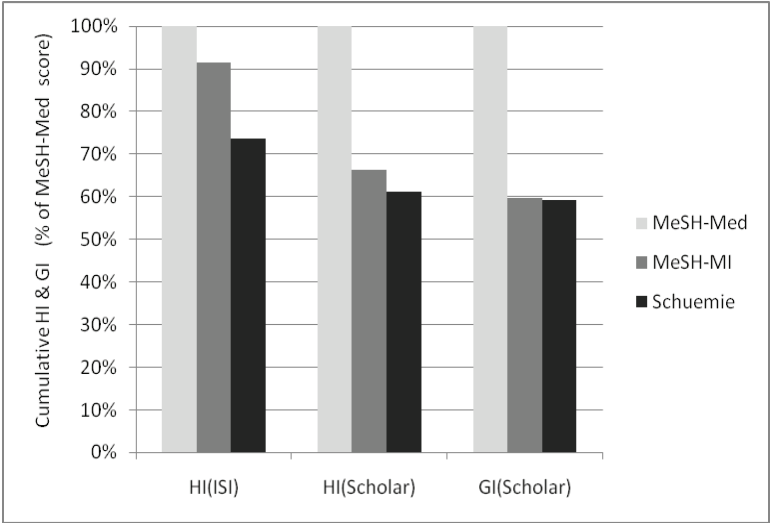
**Cumulative HI and GI of the top-25 authors of the MI-related corpora relative to the MeSH-Med corpus in percent**.

As in the case of the average scores of journal-related indices, the cumulative HI or GI values of the top-25 authors of the MI-related corpora were benchmarked against the MeSH-Med corpus (Figure 4). Compared to the journal-related indices (see above) the distance to the benchmark did not vanish, but was manifestly reduced: the scores range between 91% and 59% of the benchmark.

The relative differences between the MI-related corpora were: 24%, 8%, and 0.7% for HI(ISI), HI(Scholar) and GI(Scholar), respectively. Thus, in contrast to the new journal-related indices the author-related indices did not cause different effects on MeSH-MI and Schuemie.

In general, HI(Scholar) was greater than the HI(ISI) (see Appendix 5, Table 5). As expected from the similar definitions, differences between HI and GI are small. In the following we therefore concentrate on HI.

### Productivity rank versus HI

**Figure 5:**
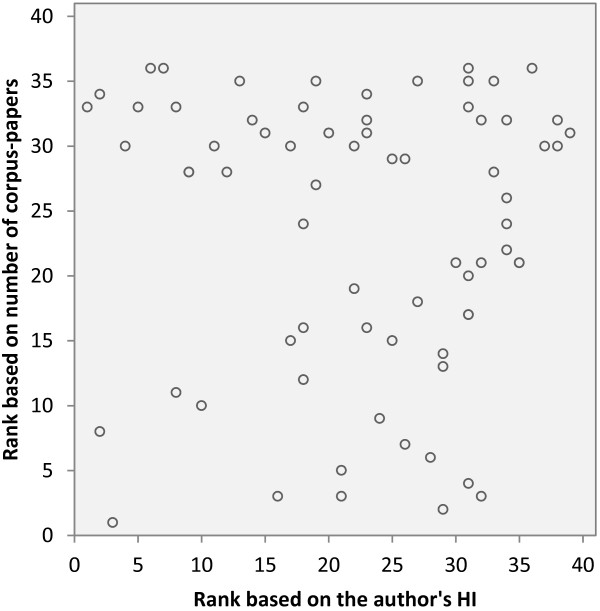
**Scatter plot of the ranking based on the number of corpus papers published by an author (productivity based ranking) and the ranking based on the authors' HI(ISI)**. The diagram includes all distinct entries (n = 72) of the top-25 most prolific authors of the three journal sets (MeSH-MI-, MeSH-Med-, Schuemie corpus).

The original ranking of the top-25 author lists based on the number of papers published in journals of the corpus (corpus-related productivity) was compared to the ranking induced by the HI(ISI) for all distinct authors named in the top-25 lists (n = 72). Visible inspection of the scatter plot (Figure 5) does not reveal any correlation. The Spearman rank correlation coefficient (rho = -.026, p = .827) indicates no significant dependence between the top-25 ranking and the ranking induced by the HI.

### Correlation between HI based on ISI Web of Science vs. Google Scholar citation data

**Figure 6:**
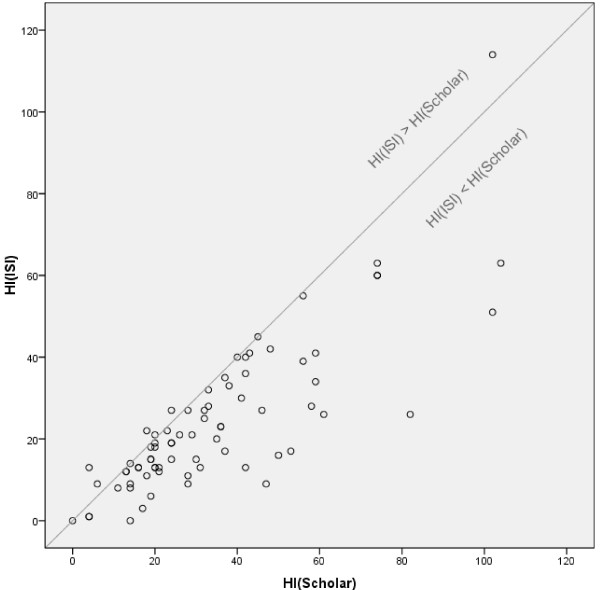
**Scatter plot of the HI(ISI) vs. the HI(Scholar)**. The diagrams show the HI(ISI) vs. the HI(Scholar) for all distinct top-25 authors (n = 72) of the three journal sets (MeSH-MI-, MeSH-Med-, Schuemie corpus).

For all distinct top-25 authors of the three journal corpora (n = 72) the correlation between HI(ISI) based on the ISI/Thompson Reuters citation data and HI(Scholar) based on Google Scholar was tested. Visual data inspection (Figure 6) and the test showed a two-tailed significant correlation (Spearman's rho: .787). Additionally, HI(Scholar) and GI(Scholar) were strongly related (significant correlation), as was expected from the definition of the two measures.

## Discussion

To the best of our knowledge, there exists no prior study which similarly investigated the changes of the bibliometric visibility of MI induced by the new indices. DeShazo et al. showed that over the last 20 years the publication output of MI outperformed the average growth of journals indexed for Medline [1]. Falagas et al. compared the JIF and the SJR in general: according to them, the SJR has a better coverage of the citation database (Scopus) [21]. This general statement was revisited by our study in the special case of MI journals. Recently, a comprehensive classification of bibliometric measures based on a principal component analysis was published [22]. The analysis of 39 established and proposed bibliometric measures yielded that (i) scientific impact "cannot be adequately measured by any single indicator, (ii) JIF should no longer be considered "the 'golden standard' of scientific impact". This is confirmed by our study.

### Definition of the corpora

The similarity analysis and the inspection of the MeSH profiles yielded a low overlap between the two MI related corpora, which challenges the approach used to define them. Nonetheless, the four criteria (Methods C) ensure that the corpora (or at least their principles of choice) were defined and accepted independently and have been considered as adequately representing MI in the scientific discussion. As shown by the MeSH-profiles, the papers of the MeSH-MI corpus frequently address aspects of bioinformatics (16 of 23 MeSH terms specific for these papers were concepts of molecular biology). The MeSH-MI corpus establishes a strong link between Medical Informatics and Bioinformatics focusing on methods and computer applications in molecular biology and translational medicine. Papers published in journals of the Schuemie corpus are multiply assigned to MeSH-subconcepts of "Medical Informatics" addressing e.g. clinical information processing. Thus, we favor the view that these corpora are not ill-defined sets, but different perspectives, found in an open and lasting discussion about the scope of MI.

### Thread of marginalization

The bibliometric visibility of MI never comes up to the visibility of certain medical fields: Similar to top-ranked journals in biology top-ranked medical journals reach a JIT value even higher than "Nature" (34.5) or "Science" (29.7) (e.g." CA - a cancer journal for clinicians", "The New England Journal of Medicine", "Annual Review of Immunology", "Physiology Review"; all scoring above 37) [7]. As shown by applying the selection criteria of the MeSH-MI corpus, these journals almost never address MI-related topics and therefore do not contribute to the visibility of MI. Apart from considering single high-scoring journals, the median JIF scores of journal categories published regularly by the ISI Web of Science can be compared. Here MI ranges among smaller medical fields (e. g. dermatology, ophthalmology, pediatrics), where top-rating medical categories (e. g. endocrinology, rheumatology, cell & tissue engineering) reach a median JIF nearly twice as high. With respect to indices rating authors the biomedical fields are known to achieve higher HI other domains e.g. physics [16]. DeShazo et al. stated an increasing visibility of MI based on data of a twenty year period (1987-2006) [1]. The statement is based on a) a markedly increasing number of MI-related articles, b) the growing number of MI-related journals, c) the trend of MI-related papers being more frequently published in high-JIF-scoring journals. While the last argument seeming to be sound, a) and b) could be questioned - given the trend of a rapidly growing number of publications in general. The possible influence of the new bibliometric indices on the visibility is not discussed in [1].

This article revisited the relatively low visibility of MI compared to medical fields in the special case of two carefully defined MI-related corpora compared to a corpus focused on Internal Medicine. The low scores of the Schuemie corpus indicate that research addressing classical MI topics suffers most from low visibility. Considering the effect of the new bibliometric indices the Schuemie corpus cannot profit from any improvement. On the contrary, the field would be almost marginalized, if the ES gained more influence in future.

But, the study has revealed some aspects providing starting points for promoting the visibility of MI (and a more optimistic view) as well:

### Interdisciplinarity pays

The MeSH-MI corpus, profits from the transition to new indices. This effect cannot be ascribed to the better index coverage, because the average scores eliminate the influence of missing index data. The MeSH-MI corpus represents MI research directly integrated into other biomedical fields. A good publication strategy would therefore read: "Go to (and publish with) the physician in order to survive a potential ES-epidemic".

### Competition on the "bibliometrics market" may help

For decades, the role of ISI/Thompson Reuters as the main provider of bibliometric indices was quasi monopolistic. Now, Scopus is considered a serious competitor and free journal-ranking tools are available [4,5,21]. As this study shows, Scopus improves the database coverage of MI corpora. Thus, indices based on Scopus (HI(SCI), SRJ) are more representative for MI and improve its bibliometric visibility compared to indices based on ISI/Thompson Reuters data (JIF, ES, AIS). Consequently, MI will profit, if Scopus-based indices gain further influence as predicted by Falagas et al. [21].

### Assessment of authors and institutions should rely on HI

Although the JIF is by definition a journal-ranking measure, it is still extensively used to assess individuals or institutions - a fact which has attracted a lot of criticism [6,7]. The author-related HI provides a better alternative: The relative independence of corpus-related productivity and HI shown in this study affirms that HI merely indicates the sustainable scientific impact than the quantitative output in a given domain. This supports the claim that HI is an adequate measure of scientific quality [16,17]. By and large, the HI based on ISI citation data is in accordance to the HI based on web publications, yielding an unexpected connection between the visibilities based on web publications vs. journal databases. Not surprisingly, the MI-authors did not fully meet the benchmark set by medical authors. But, compared to the journal-related indices, the disproportions were manifestly reduced. This fact was surprising, because it is known that the HIs of (bio-)medical researchers are manifestly higher compared to other domains [16]. Obviously, prolific researchers in MI succeed in gaining a degree of attention and reputation comparable to that of their medical counterparts - possibly by publications in high-impact journals of more general interest.

### Limitations

When retrieving paper counts or authors, we did not exclude special types of publication. In one case this lead to the inclusion of a journals' staff writer (Dimick C) into the top-25 author list. Therefore, MeSH-MI was corrected by considering the 26^th ^author of the ranking instead.

This study is not based on random samples of journals, but gives a complete survey for the journals included. Thus, a significance analysis was neither feasible nor necessary: the aggregate scores were applied as descriptive means to characterize the total bibliometric visibility.

The HI typically suffers from its reliance on name strings: although information on the authors' affiliation and additional surnames were taken into account, citations of different authors sharing the same name may contribute to the HI (this problem especially arose for five Chinese names). Another source of incorrect HI is authors changing their names e.g. by marriage. This problem must be addressed in the future by implementing unique personal/institutional IDs. A first attempt has already been implemented in Scopus [23].

## Conclusions

The visibility of Medical Informatics (investigated here as an example of a small, multidisciplinary field) is specifically changed by the newly established journal-related indices: the core of classical MI research (represented by the Schuemie corpus) remains on a 10%-level of visibility compared to the medical benchmark. In contrast, MI research in the context of interdisciplinary projects generally profits from the new indices.

As for the author-related indices, the HI proved to shed a different light on the research activity of a field and provides a far more adequate means to assess individuals than journal-related indices. Interestingly, the HI impact of an author can well be estimated based on Web publications (Scholar publication data).

The public availability of bibliometric information has dramatically improved in the last years. This could result in a further increased misuse, but also (and hopefully) in a broader dissemination and increased understanding of bibliometric approaches [7].

## Appendices

### APPENDIX 1: Bibliometric tools

In total, five biometric tools have been used for this study. They can be described briefly as following

• *GoPubmed *can be used to analyse the results of a PubMed query. It categorizes the publications retrieved by associated MeSH terms and produces comprehensive statistics including author and journal rankings.

• *ISI Web of Science *is the commercial web portal of the bibliographic and bibliometric databases of Thomson Reuters Corp., New York, NY, USA, and the primary source of JIF, ES and AIS.

• *QuadSearch *provides access to the HI and GI based on the citation data of Google Scholar, a Google provided service to search for scholarly literature across many disciplines and sources, including theses, books, abstracts and articles (HI(Scholar), GI(Scholar)).

• *SCImago Web Portal *provides free access to the bibliometric indices SRJ and HI(SCI) derived from the Scopus citation data. In 2004, the bibliographic database Scopus, which is restricted to commercial use, was funded by an international consortium led by Elsevier B.V., Amsterdam, to directly compete with the ISI Web of Science.

• *NCBI "Journals" Database *is provided by the National Center for Biotechnology Information, National Library of Medicine (NLM), National Institutes of Health (NIH), Bethesda, MD, USA, and allows standardization of journal names and abbreviations, which are not used uniformly in the literature.

### APPENDIX 2: Defining the Schuemie corpus

In order to define a corpus of MI-related journals Schuemie et al. apply the following approach: The procedure starts with an initial set of journals, given by the journals assigned to the ISI category "Medical Informatics". For each journal an n-gram profile is calculated using the titles and abstracts of the articles published by the journal. Basically an n-gram profile is constructed by counting the occurrences of sequences of n characters in a given string (e. g. the string "This_is" yields the following bigram (2-gram) profile: "Th":1, "hi": 1, "is":2, "s_": 1, "_i":1). Instead of absolute counts, n-gram profiles are often constructed from normalized weights measuring the information content of the respective character sequences. The authors use normalized uni-, bi-, and trigrams. For a given (lexicographical) order of the n-grams the numbers of occurrences form a vector, which can be compared with the respective vector of a different string or text in order to calculate a similarity score. Here, the normalized scalar product (i.e., the angle between the two vectors) quantifies the similarity (see Appendix 4). Given a test set *T *and a seed subset *S *of journals a result set of similar journals (R) is calculated by the following iterative procedure: For each journal of *T*, the sum of similarity scores between the journal and all journals of *S *is calculated. The journals are ranked according to sum scores. All journals having equal or better score than the lowest ranking seed journal form a new seed set and the procedure is iterated.

### Appendix 3: Overview of the journal corpora

The following table provides an overview of the journal corpora addressed in this study (MeSH-MI, Schuemie, MeSH-Med). Journals belonging to more than one corpus are highlighted (bold face font).

### APPENDIX 4: Vector space distance (Cosine Similarity) of MeSH Profiles

Let the union of the MeSH-profiles of given corpus contain n different MeSH terms. Then, the respective term frequency rates (i.e., the frequency with which a given MeSH term was assigned to the papers of the corpus) form an n-dimensional vector. For each pair of corpora, the normalized scalar product of these vectors - essentially the cosine of the angle enclosed - is used as a similarity measure ranging from 0 (least similar) to 1 (most similar). The measure is referred to as cosine similarity or vector space distance. A value of 0.2 - as obtained when comparing Schuemie vs. MeSH-Med corpus - corresponds to an angle of 78° (where 90° indicates completely different profiles). The following simple example would yield the same value: Two corpora are characterized by profiles of 10 terms each, the term frequency never exceeds 1, and the profiles have only 2 terms in common (i.e. both profiles contain a total of 18 distinct terms).

### APPENDIX 5: HI achieved by the top-25 authors

The following table 5 shows the sums of the HI achieved by the top-25 authors of MeSH-MI, Schuemie, and MeSH-Med corpus, respectively. The values in parenthesis are the mean HI of the top-25 authors and the respective standard derivation.

**Table 5:** HI achieved by the top-25

	MeSH-MI	Schuemie	MeSH-Med
HI(ISI)	624 (25 ± 15.5)	503 (20.1 ± 11.1)	683 (27.3 ± 25.5)
HI(Scholar)	780 (31.2 ± 20.5)	694 (27.8 ± 15.4)	1135 (45.4 ± 26.7)

## Competing interests

The authors confirm that there are no financial or non-financial competing interests (political, personal, religious, ideological, academic, intellectual, commercial or any other) to declare in relation to this manuscript.

## Authors' contributions

CS carried out the collection and analysis of the bibliometric data and drafted the manuscript. TMD inspired the investigation of the effect of the new bibliometric indices on the visibility of Medical Informatics, formulated the research hypotheses and deeply supported the preparation and organization of the manuscript. KS contributed substantially to the correlation analysis. All authors read and approved the final manuscript.

## Pre-publication history

The pre-publication history for this paper can be accessed here:

http://www.biomedcentral.com/1472-6947/11/24/prepub
